# Machine-Learning Approaches for Classifying Haplogroup from Y Chromosome STR Data

**DOI:** 10.1371/journal.pcbi.1000093

**Published:** 2008-06-13

**Authors:** Joseph Schlecht, Matthew E. Kaplan, Kobus Barnard, Tatiana Karafet, Michael F. Hammer, Nirav C. Merchant

**Affiliations:** 1 Computer Science Department, University of Arizona, Tucson, Arizona, United States of America; 2 Arizona Research Laboratories, University of Arizona, Tucson, Arizona, United States of America; King's College London, United Kingdom

## Abstract

Genetic variation on the non-recombining portion of the Y chromosome contains information about the ancestry of male lineages. Because of their low rate of mutation, single nucleotide polymorphisms (SNPs) are the markers of choice for unambiguously classifying Y chromosomes into related sets of lineages known as haplogroups, which tend to show geographic structure in many parts of the world. However, performing the large number of SNP genotyping tests needed to properly infer haplogroup status is expensive and time consuming. A novel alternative for assigning a sampled Y chromosome to a haplogroup is presented here. We show that by applying modern machine-learning algorithms we can infer with high accuracy the proper Y chromosome haplogroup of a sample by scoring a relatively small number of Y-linked short tandem repeats (STRs). Learning is based on a diverse ground-truth data set comprising pairs of SNP test results (haplogroup) and corresponding STR scores. We apply several independent machine-learning methods in tandem to learn formal classification functions. The result is an integrated high-throughput analysis system that automatically classifies large numbers of samples into haplogroups in a cost-effective and accurate manner.

## Author Summary

The Y chromosome is passed on from father to son as a nearly identical copy. Occasionally, small random changes occur in the Y DNA sequences that are passed forward to the next generation. There are two kinds of changes that may occur, and they both provide vital information for the study of human ancestry. Of the two kinds, one is a single letter change, and the other is a change in the number of short tandemly repeating sequences. The single-letter changes can be laborious to test, but they provide information on deep ancestry. Measuring the number of sequence repeats at multiple places in the genome simultaneously is efficient, and provides information about recent history at a modest cost. We present the novel approach of training a collection of modern machine-learning algorithms with these sequence repeats to infer the single-letter changes, thus assigning the samples to deep ancestry lineages.

## Introduction

Genetic variation on the non-recombining portion of the Y chromosome (NRY) has become the target of many recent studies with applications in a variety of disciplines, including DNA forensics [1],[2], medical genetics [3], genealogical reconstruction [4], molecular archeology [5], non-human primate genetics [6], and human evolutionary studies [7]–[9]. Two extremely useful classes of marker on the NRY include microsatellites or short tandem repeats (STRs) and single nucleotide polymorphism (SNPs) [7]. STRs consist of variable numbers of tandem repeat units ranging from 1 to 6-bp in length and mutate via a stepwise mutation mechanism, which favors very small (usually one repeat unit) changes in array length. Because high mutation rates (estimated to be 0.23%/STR/generation) in human pedigrees [10],[11] often lead to situations where two alleles with the same repeat number are not identical by descent, STRs are not the marker of choice for constructing trees or for inferring relationships among divergent human populations. Rather, the high heterozygosity of STRs makes them useful for forensic and paternity analysis, and for inferring affinities among closely related populations.

Reconstructing relationships among globally dispersed populations or divergent male lineages requires polymorphisms with lower probabilities of back and parallel mutation (i.e., lower levels of homoplasy) and systems for which the ancestral state can be determined. SNPs and small indels, with mutation rates on the order of 2-4×10^−8^/site/generation, are best suited for these purposes. Because SNPs and indels are likely to have only two allelic classes segregating in human populations, they are sometimes referred to as binary markers (we refer to both classes of marker as SNPs). The combination of allelic states at many SNPs on a given Y chromosome is known as a haplogroup. A binary tree of NRY haplogroups with a standard nomenclature system (Figure S1) has been published and widely accepted among workers in the Y chromosome field [12],[13]. This Y chromosome tree is characterized by a hierarchically arranged set of 18 arbitrarily defined clusters of lineages (clades A–R), each with several sub-clades. By typing informative sets of SNPs, it is possible to assign samples to particular clades or subclades [9],[14].

One of the challenges for geneticists is the cost and time typically needed to genotype an appropriate number of SNPs to assign a given Y chromosome to a haplogroup. Multiplex strategies to type SNPs are also difficult and require a substantial initial investment to implement [15]. STRs on the NRY (Y-STRs) offer an alternative method for inferring the haplogroup of a sample. It has been recognized for some time that STR variability is partitioned to a greater extent by differences among haplogroups than by differences among populations [16],[17]. This suggests that Y-STRs contain information about the haplogroup status of a given Y chromosome. Because many Y-STRs can be genotyped in multiplex assays, typing appropriate sets of Y-STRs could represent a cost effective strategy for classifying Y chromosomes into haplogroups. In this paper, we assess this possibility from a computational perspective and show how a suite of modern machine learning algorithms can automatically classify and predict haplogroups based on allelic data from a suite of Y-STRs. We adapt three types of classifiers based on both generative and discriminative models to this problem. When all the methods agree in tandem, we combine the classifications from each into a haplogroup assignment. This enables an automatic, high throughput analysis pipeline for determining the haplogroup of a large number of samples in a cost effective and accurate manner.

## Results and Discussion

**Figure 1:**
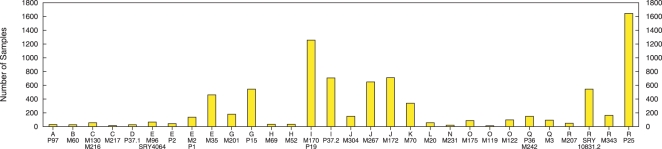
Frequency of 30 haplogroups determined by SNP-typing a geographically diverse sample of 8,414 chromosomes. This set of chromosomes, typed at 15 Y-linked STRs, was used as a ground-truth training set (see text for explanation). Haplogroups are named according to the mutation-based nomenclature [12], which retains the major haplogroup information (i.e., 18 capital letters) followed by the name of the terminal mutation that the sample is positive for (see Figure S1).

We obtained a data set collected by the Hammer laboratory that contains 8,414 globally diverse Y chromosome samples genotyped at 15 Y-STRs. The same samples were also typed with a battery of SNPs to identify the haplogroup of each sample. The SNPs typed and the resulting haplogroup tree are given in Figure S1, and the frequency of haplogroups in our data set is shown in Figure 1.

Since each sample of STR scores in our data is labeled with a haplogroup, we formalized the problem of classifying a new sample with a haplogroup as a supervised learning task and used our data set of 8,414 samples as a ground-truth set for training. In order to provide high classification accuracy, we combined the results of three disparate types of classifiers: decision trees, generative Bayesian models, and support vector machines. As we describe in Materials and Methods, each algorithm has a unique method of learning haplogroup classification from training data; none commit exactly the same type of error and combining their output yields a more robust decision [18]. Indeed, our results show that combining the classification output from these methods yields very accurate haplogroup assignment.

We compared our classification results to an informal nearest neighbor heuristic that labels STR samples with a haplogroup based on the stepwise mutation model [19]. We show that its results are not as effective as our tandem of machine learning techniques.

### Classifier Evaluation

We evaluated the performance of each classifier individually and in tandem using cross-validation on our 8,414 sample ground-truth training set, and compared the results with the nearest neighbor heuristic previously mentioned. We also performed cross-validation on publicly available data from other published research with Y-STR and haplogroup data. Finally, we tested the classification performance on the public data using our data for training. In brief, the results show the classifiers perform very well with a diverse training set and that the number of loci available in the data set is an important determining factor in their performance.

The cross-validation was accomplished by stochastically partitioning the data sets into *k* equally sized subsets, iteratively holding out each one while training on the remaining data, and then testing on the held out subset. More formally, let the ground-truth data set with *N* samples be 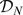. We create equally sized subsets 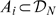 for 1≤*i*≤*k* that form a partition of 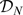, i.e.,
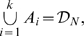
(1)

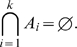
(2)We held out each subset *A_i_* of the partition and trained the classifiers on the set 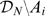. A classification test was then performed on the held out set. In practice, the subset sizes may differ by one if *N*/*k* is not integral. For our experiments we chose to use *k* = 5 folds. The cross-validation was repeated 10 iterations, each time generating a random, equal partition of the data. The performance results were finally compiled with the mean and standard error statistics.

We combined the classification output for a sample from the decision trees (J48 and PART), Bayesian models, and support vector machines into a tandem decision. The output haplogroups from each of the classifiers were compared together, and if they were in agreement, accepted, or assigned, the classification; otherwise the sample was left *unassigned* if they disagree and held-out for further analysis. Since the classifications may not always be at the same depth in the haplogroup hierarchy, Figure S1, we compared the results up to the common level in the tree and accepted the classification if it was in agreement.

In practice, an unassigned sample for the tandem approach is selected for manual, expert analysis. Experienced personnel examine the haplogroup assignment from the individual classifiers for familiar patterns. The confidence values from the classifiers may also be analyzed to resolve frequently seen disagreements. If the ambiguity cannot be resolved at this stage, SNP testing is done to ensure a correct haplogroup label. The result of the SNP test is then added to the training set to continually improve the classifiers.

For the nearest neighbor heuristic we used the *L* ^1^-norm distance metric combined with the following rules. If the sum of allele value differences between a novel sample and one in the training set was zero, it was an exact match and the novel sample was labeled with the matching sample's haplogroup. If the allele values differed by only one or two, and the samples by which it differed were all in the same haplogroup, it was considered a match resulting from a stepwise mutation and again labeled with the matching samples' haplogroup. Otherwise, the sample was left unassigned.

Table 1 shows the average overall performance of the classifiers, including tandem agreement and the nearest neighbor heuristic, for ten iterations of the 5 fold cross-validation on our ground-truth training set. The support vector machine was the best performing individual classifier with 95% accuracy. The performance of the Bayesian classifier and the decision trees was very comparable. The results for the tandem strategy show that of all the samples we attempted to classify, 86% were in agreement, and that almost 99% of those predictions were correct. Furthermore, the 14% unassignment rate of the tandem approach was much lower than the 26% of the nearest neighbor heuristic.

**Table 1:** Average classifier performance for cross-validation on our 8,414 sample ground-truth training set (see text for experiment details).

	Percent Correct of Assigned		Correct		Incorrect		Unassigned	
	Mean	SE	Mean	SE	Mean	SE	Mean	SE
Tandem	98.8	0.1	1426.1	1.8	17.9	0.7	238.8	1.9
SVM	95.0	0.1	1598.5	1.2	84.3	1.2		
J48	92.7	0.1	1559.5	1.5	123.3	1.5		
PART	92.5	0.1	1556.8	1.6	126.0	1.6		
Bayes	91.5	0.1	1540.2	1.5	142.6	1.5		
Nearest	98.3	0.2	1227.3	2.5	21.6	0.5	433.9	2.6

Support vector machines has the highest individual accuracy. J48 and PART are decision tree classifiers. Combining the classifiers together into the tandem strategy boosts the performance to a very high accuracy while maintaining a much lower unassignment rate than the nearest neighbor heuristic.

**Figure 2:**
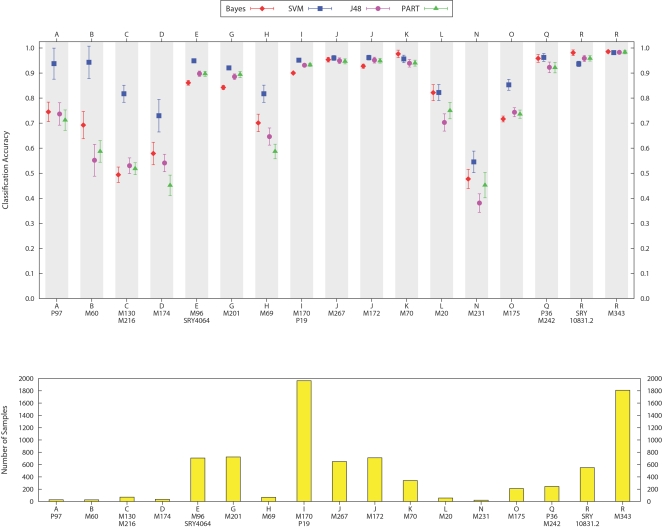
Average accuracy of each classifier per haplogroup for cross-validation on the ground-truth training set. Standard error bars are shown for each point. The lower panel shows the frequency of the 8,414 samples among haplogroups. Support vector machines has the best overall performance, especially in the case of haplogroups with a smaller number of samples in the training data.

The average accuracy for each of the classifiers per haplogroup is shown in the top panel of Figure 2, and the haplogroup frequency of the training data is below it in the bottom panel. It is clear from the figure that the accuracy of classification for a particular haplogroup is dependent on its frequency in the data. We also observe that the support vector machines perform the best, particularly in cases where training data for a haplogroup is most sparse. We believe that more training data from sparse groups, such as A, B, C, D, H, and N would improve the results to similar levels of more well represented haplogroups such as I, J, and R.

**Figure 3:**
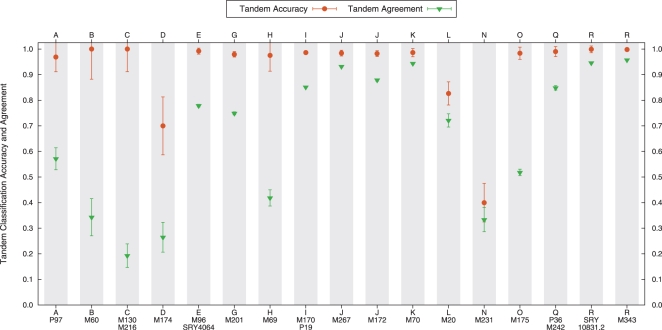
Average accuracy of the tandem approach for cross-validation on the ground-truth training set. The average proportion of samples with agreement for all four classification methods is also shown. The haplogroups with the highest rate of tandem disagreement have a low representation in the training data.

The classification accuracy under the tandem approach was very high. Figure 3 shows the performance for each haplogroup when all the classifiers agreed. While not all of the classifiers agreed in their output in all cases, we observe from the results in Figure 3 and Table 2 that the rate of agreement was very low mostly in haplogroups with low representation in the data. Again, as we continue to increase the size and diversity of the training set, we expect that the level of agreement in the tandem approach will continue to improve.

**Table 2:** Average haplogroup assignment accuracy after cross-validation of the ground-truth training data for the tandem approach when all classifiers agree.

Haplogroup	Correct		Incorrect		Unassigned	
	Mean	SE	Mean	SE	Mean	SE
A- P97	3.1	0.2	0.1	0.0	2.4	0.2
B- M60	1.2	0.1	0.0	0.0	2.3	0.3
C- M130,M216	1.6	0.1	0.0	0.0	6.7	0.4
D- M174	0.7	0.1	0.2	0.1	2.5	0.2
E- M96,SRY4064	111.4	1.3	0.9	0.1	31.9	0.8
G- M201	114.9	1.2	2.4	0.2	39.3	0.9
H- M69	4.0	0.3	0.1	0.0	5.7	0.3
I- M170,P19	335.7	2.2	4.7	0.4	59.4	1.1
J- M267	125.4	1.5	2.0	0.2	9.3	0.4
J- M172	128.4	1.5	2.3	0.2	18.0	0.7
K- M70	62.9	1.0	0.9	0.1	3.8	0.3
L- M20	6.2	0.3	1.3	0.2	2.9	0.3
N- M231	0.6	0.1	0.9	0.2	3.0	0.2
O- M175	24.0	0.6	0.4	0.1	22.7	0.6
Q- P36,M242	40.5	0.8	0.4	0.1	7.3	0.4
R- SRY10831.2	100.0	1.3	0.1	0.0	5.7	0.3
R- M343	346.7	1.7	0.6	0.1	15.5	0.6

The far right column gives the number of samples with an unassigned tandem classification—all four methods did not agree.

**Figure 4:**
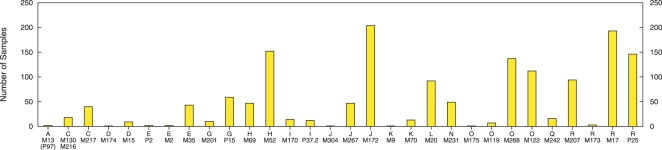
Frequency of 30 Y chromosome haplogroups inferred from a previously published sample of 1,527 Asian Y chromosomes. The samples were typed with 9 Y-STRs and a battery of Y-linked SNPs. Haplogroup frequencies are statistically significantly different from those in our ground-truth training set (Figure 1). Haplogroups are named according to the mutation-based nomenclature [12], which retains the major haplogroup information (i.e., 18 capital letters) followed by the name of the terminal mutation that the sample is positive for (see Figure S1).

In addition to testing the performance of the classifiers on our 15-locus data set, we also tested them on published STR data collected from West, South and East Asian populations [20],[21]. The combined public data sets have 1,527 samples of 9 loci at DYS394, DYS388, DYS389-I, DYS389-II, DYS390, DYS391, DYS392, DYS393, and DYS439. Figure 4 shows the frequencies of Y chromosome haplogroups in this sample. We performed two types of experiments with this data. We first looked at performance using the public data both for training and testing with a 5 fold cross-validation. We then used our ground-truth data set restricted to the 9 applicable loci as training data, and tested the performance on the entire public data set.

**Figure 5:**
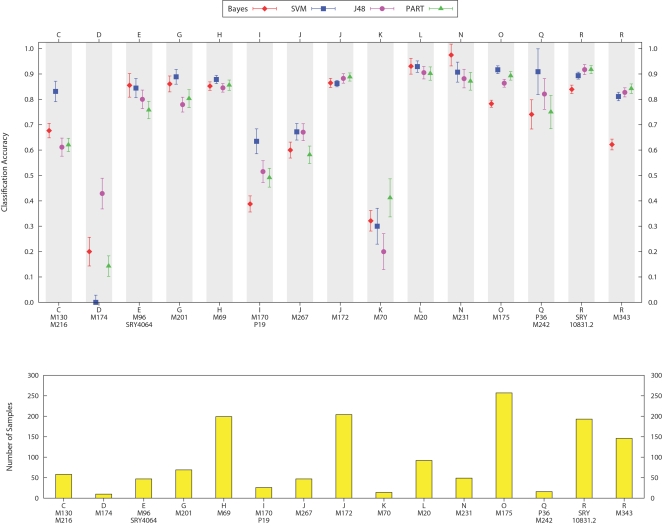
Average accuracy of each classifier per haplogroup for cross-validation on the 9-locus public STR data. Standard error bars are shown for each point. The lower panel shows the frequency of haplogroups in the 1,527 sample public data set.

**Figure 6:**
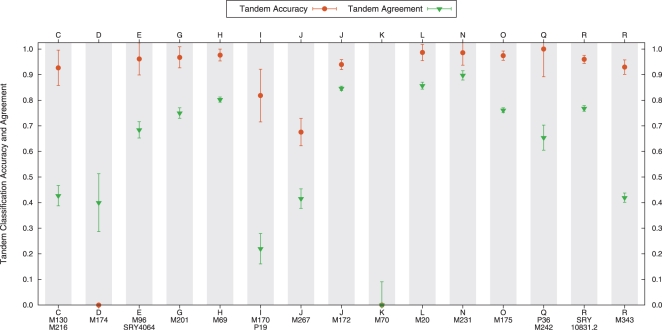
Average accuracy and agreement of the tandem approach for cross-validation on the 9-locus public STR data.

As before when testing the classifiers on our ground-truth data, we ran 10 iterations of five fold cross-validation on the public data. Table 3 gives the averaged results. In order to provide a meaningful comparison across the two data sets, the table also shows cross-validation results on the 9-locus subset of our data; six of the loci are not shared by both sets and may affect discriminative abilities of the classifiers. We observe that the classification accuracy between the two data sets is comparable. Indeed, the cross-validation on the public data has slightly better results. Figure 5 and Figure 6 show the average per haplogroup classification accuracy for cross-validation on the public data.

**Table 3:** Comparison of classifier performance across two data sets.

9-Locus Public Data								
	Percent Correct of Assigned		Correct		Incorrect		Unassigned	
	Mean	SE	Mean	SE	Mean	SE	Mean	SE
Tandem	95.6	0.5	208.6	1.1	9.5	0.3	87.3	1.1
SVM	87.1	0.2	265.9	0.7	39.5	0.7		
PART	84.3	0.3	257.6	0.8	47.8	0.8		
J48	84.3	0.3	257.5	0.8	47.9	0.8		
Bayes	79.0	0.3	241.3	0.9	64.1	0.9		
Nearest	92.6	0.4	212.8	1.0	17.0	0.6	75.6	0.8

9-Locus Ground-Truth Data								
	Percent Correct of Assigned		Correct		Incorrect		Unassigned	
	Mean	SE	Mean	SE	Mean	SE	Mean	SE
Tandem	92.4	0.2	1,100.2	2.4	90.0	1.1	616.7	2.4
SVM	82.4	0.1	1,386.1	1.9	296.7	1.9		
PART	81.1	0.1	1,364.2	1.9	318.6	1.9		
J48	81.2	0.1	1,366.5	1.9	316.3	1.9		
Bayes	76.9	0.1	1,293.4	2.1	389.4	2.1		
Nearest	91.8	0.4	618.6	2.9	55.6	1.1	1,008.7	3.3

The top table shows the average classifier performance for cross-validation on the 9-locus public STR data. The bottom table is the performance for the same test, but on a 9-locus subset of our ground-truth training data. While overall performance is lower than the 15-locus cross-validation test on our ground-truth data (Table 1), the two data sets perform similarly here, indicating that increasing the number of markers in the data set can significantly improve performance.

Compared with the earlier cross-validation results on our ground-truth data (Table 1), the 9 Y-STR subset has a much lower performance than the original 15 Y-STR set. This implies that the 6 excluded markers contribute to a non-negligible increase in performance. Thus, if the public data set had these additional markers, we expect that its accuracy under cross-validation would also improve.

We tested haplogroup classification for the public STR data using classifiers trained with the 9-locus subset of our data set. The classification accuracy results are reported in Table 4. Figures S2 and S3 show the average per haplogroup performance. Although the tandem approach still out-performed the nearest neighbor method, the overall performance shows a decrease in accuracy. We believe the performance is lower for two reasons: as we have already shown, training with 9 versus 15 Y-STRs substantially reduces the accuracy of classification (an almost 7 point reduction for the tandem approach when contrasting Table 1 with the lower panel of Table 3); and the origins of the samples in the public data sets are from populations that are not as well represented in our data set.

**Table 4:** Classification results for the 9-locus public Y-STR data.

	Percent CoA	Correct	Incorrect	Unassigned
Tandem	83.9	732	140	655
SVM	67.2	1,026	501	
PART	70.5	1,077	450	
J48	70.5	1,076	451	
Bayes	62.8	959	568	
Nearest	81.9	398	88	1,041

A 9-locus subset of our ground-truth data was used to train the classifiers (Percent CoA = % Correct of Assigned).

### Conclusion

In this paper we have shown that by using machine learning algorithms and data derived only from a set of Y-linked STRs, it is possible to assign Y chromosome haplogroups to individual samples with a high degree of accuracy. We note that the number of Y-STRs used has a significant impact on the accuracy of haplogroup classification.

Our classification software provides a single turnkey interface to a tandem of machine learning algorithms. It is extensible in that other high-performing classification algorithms can be added to it when they are developed. We have made the software freely available to use for non-commercial purposes and posted it online at http://bcf.arl.arizona.edu/haplo.

Future work could focus on identifying an optimal set of Y-STRs to obtain the highest accuracy of haplogroup classification. Our preliminary results (data not shown) suggest that different Y-STRs are informative for different haplogroups. Additional work should help to better understand the properties that make different Y-STRs more or less informative for proper haplogroup assignment.

We have assumed in our Bayesian model that the Y-STR loci are statistically independent given the haplogroup. While we have observed good performance for this model, it most likely does not reflect the true relationship among loci. As more information about loci linkage becomes available and our ground-truth data set continues to expand, we could relax this assumption and begin to include such dependencies.

Our Bayesian model assumes that Y-STRs are statistically independent conditioned on the haplogroup. While we observe good performance using this model, this assumption is not realistic given the lack of crossing over among the Y-linked STRs used in our analysis. On the other hand, Y-STRs mutate independently in a stepwise fashion, which may cause particular Y-STRs to be effectively unlinked on some haplogroup backgrounds. As more information about linkage becomes available and our ground-truth data set continues to expand, we may be able to include such information to improve our model.

The software system can be effectively used to construct high throughput SNP test panels, particularly in the case of platforms that restrict the number of SNPs accommodated per panel. Given a corpus of STR data, the classifiers can identify a collection of candidate SNP sites to be placed on the panel to provide maximum coverage over potential haplogroups in a population. In this way the software provides a cost-effective first step in a multi-level process for deep haplogroup identification by facilitating targeted SNP testing.

## Materials and Methods

The 15 Y chromosome STR loci used in this study are: DYS393, DYS390, DYS394 (both copies when duplicated), DYS391, DYS385a, DYS385b, DYS426, DYS388, DYS439, DYS389 I, DYS389 II, DYS392, DYS438, DYS457. These loci are commonly used in the fields of population genetics, forensic science, and commercial genealogical testing [22]. The STR loci were amplified in two multiplex PCR reactions. The products of these reactions were mixed and analyzed on an Applied Biosystems 3730 capillary electrophoresis instrument.

The SNP and STR data for samples utilized to construct the training data set and models for this study were acquired and analyzed over an extended period of time by the Hammer laboratory. The SNPs were identified using a variety of techniques including: DNA sequencing, allele specific PCR scored by agarose electrophoresis, PCR and restriction digest, and TaqMan assays. A test panel comprising the SNPs in Figure 1 was developed and validated for use on a Beckman Coulter SNPStream instrument [23]. This instrument permits simultaneous testing for all SNPs represented on the panel for a given sample. Novel samples utilized for testing and validation of the models were STR tested and processed on the SNPStream instrument to verify the predicted SNP assignments.

What follows is a brief description of the classifiers we used and how each was adapted and extended to the haplogroup assignment problem. We first introduce some notation shared among all classifier descriptions. Let *L* be the number of analyzed Y-STRs and *G* be number of haplogroups under consideration. Denote the ground-truth data set of *N* samples by 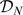. Each sample in the set comprises a tuple of haplogroup index and STR alleles 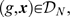 where 1≤*g*≤*G* and **x** = (*x*_1_,…,*x_L_*). Where applicable, let **X** = (*X*_1_,…,*X_L_*) be random variables taking alleles from the *L* loci on the Y chromosome.

### Decision Trees

In a decision tree classifier, we learn a set of rules for separating samples into hierarchical classification groups according to locus and allele values. The internal nodes of the tree are comprised of locus tests for specific allele values and the terminal nodes represent haplogroup classification. The set of tests from the root node in the tree to a terminal node is the classification rule for a haplogroup. The tree is constructed from a set of training data 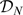 using the C4.5 algorithm [24], which hierarchically selects loci that best differentiate the training data into haplogroups.

The locus tests are constructed using a measure of information gain, which is based on information entropy [25]. The entropy of a random variable quantifies its randomness or uncertainty. In the case of haplogroups, entropy indicates how much diversity there is in the sample set.

Let *n_g_* be the number of samples in haplogroup *g*. The entropy for *G* haplogroups over the data set 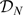 is defined as
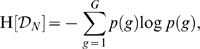
(3)where *p*(*g*) = *n_g_*/*N* is the probability of the *g* ^th^ haplogroup in the data set. Thus, higher entropy suggests higher diversity and a more uniform frequency of haplogroup representation in the sample set.

Knowing the allele value of a locus may affect the entropy of the data; additional information either does not change or decreases the entropy. When a particular allele at the *i* ^th^ locus is known, the conditional entropy is given by
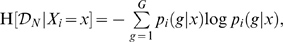
(4)where *p_i_*(*g*|*x*) is the probability the *g* ^th^ haplogroup has allele *x* at locus *i*. Let 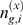 be the number of samples with the latter characteristic and 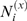 be the total number of samples in the data with allele *x* at locus *i*. Then *p_i_* is defined as
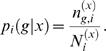
(5)


We obtain a general conditional entropy for each locus by marginalizing out the allele values. This is equivalent to computing a weighted average of Equation 4, where the weights are given by the probability of each allele.
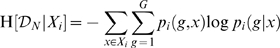
(6)

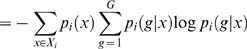
(7)

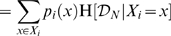
(8)where *p_i_*(*x*) is the probability of allele *x* at the *i* ^th^ locus over all samples in the data. It is given by
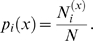
(9)The general conditional entropy in Equation 8 tells us how much Y-STR allelic variation is associated with a given haplogroup. A lower value indicates the allele values at the locus explain or predict the haplogroup well. This leads to the concept of information gain.

The difference in variation among haplogroups when a Y-STR allele is both known and unknown is the information gain. It is a measure of how well a locus explains haplogroup membership. Formally, it is defined for the *i* ^th^ locus in the data as
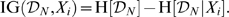
(10)The information gain will always be non-negative, since 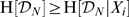 for all loci.

**Figure 7:**
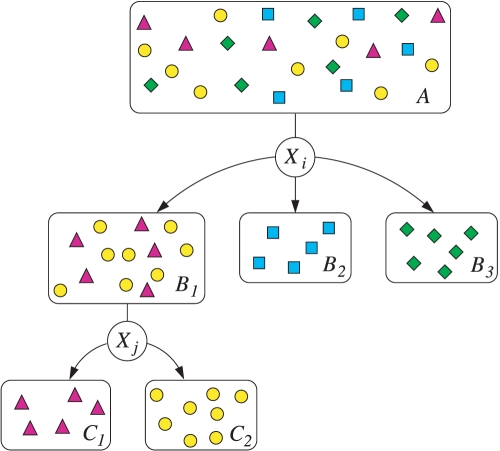
Test creation process for decision trees. Samples from four haplogroups in data set *A* are passed through locus-specific allele test conditions at each branch of the decision tree. The test for locus *X_i_* is chosen so that *i* = arg max*_l_*{IG(*A*, *X_l_*)} and *X_j_* so that *j* = arg max*l*{ IG(*B*_1_, *X_l_*)}.

Given the data set 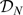, we trained a decision tree by hierarchically computing the information gain for each Y-STR. A branch in the tree is created from the locus yielding the maximum gain. The branch is a test created using the selected locus to divide the data set into subsets grouped by haplogroup and (possibly shared) allele values. Tests at lower levels of the tree are constructed from these subsets in a similar fashion. Once all the samples in a subset are in the same haplogroup, a terminal leaf on the tree is created, which represents a classification. Figure 7 illustrates this process. To classify a new sample, we begin at the root and evaluate the locus tests down the tree with its allele values until a terminal node, representing the classified haplogroup, is reached.

The general decision tree approach has some limitations, including overfitting by creating too many branches and locus bias. The former can be handled by introducing thresholds or other heuristics for the amount of information gain required to create a branch. The latter is a more fundamental problem of the approach; by definition, the information gain favors Y-STRs taking many different allele values. We used the PART and J48 implementations [26] of the decision tree algorithm in order to mitigate the effects of some of these limitations.

### Bayesian Model

In the non-parametric Bayesian model, we define a posterior distribution over the haplogroups conditioned on observed allele values. The posterior is expressed as the normalized product of the data likelihood and model prior. For a given sample of allele values, the posterior gives a probability for each haplogroup it could belong to. It is defined as
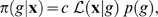
(11)where *c* is a normalization constant, and *p*(⋅)is the prior probability over the haplogroups. The likelihood function, 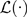, is a measure of how likely it is that haplogroup *g* generated sample **x**.

The fundamental assumption of our naive Bayes model is the independence of the Y-STRs **X** = (*X*_1_,…,*X_L_*), given the haplogroup *g*. A number of possible sources of dependency exist that could weaken the validity of this assumption. For example, Y-STRs are located on the same chromosome and physically linked, which introduces co-inheritance and the possibility of statistical linkage over short time scales. However, such statistical relationships are not sufficiently understood to be easily incorporated. Furthermore, attempting to exploit them through direct use of our ground-truth training data is not feasible because the relatively large number of dimensions [15] would require far more data. In short, the simplifying conditional independence assumption makes using our data tractable. Interestingly, the accuracy of naive Bayes classifiers is not tightly linked to the validity of this assumption [27],[28], which directly affects the accuracy of the posterior computation, but only indirectly affects the ability of the model to distinguish between groups on real data. In practice, the naive Bayes classier often performs well, and thus we chose to empirically study it for haplogroup identification.

**Figure 8:**
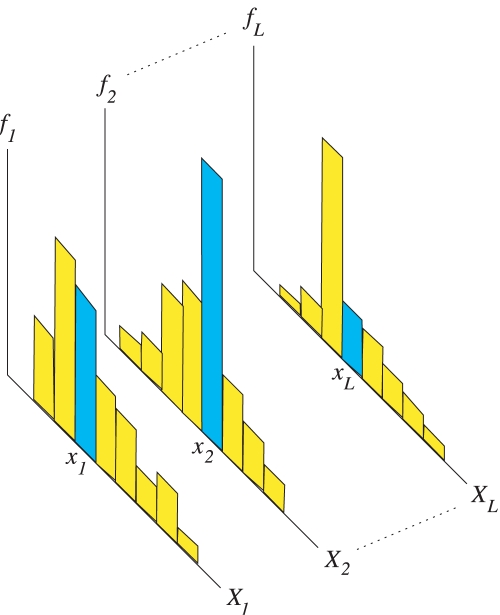
Bayesian likelihood construction and evaluation. For each haplogroup, the density functions *f*_1_,…*f_L_* are constructed as normalized histograms from the training data 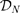. Given a sample x = (*x*_1_,…*x_L_*), its likelihood under a haplogroup is the product of its evaluated locus bin frequencies.

Mathematically, the independence assumption leads to defining the likelihood as a product over each Y-STR density function,
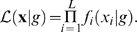
(12)We estimated the density functions *f_i_*(⋅)using histograms constructed from the data. For each Y-STR and haplogroup, we created a normalized histogram from the training data 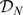 with bins corresponding to the different allele values the Y-STRs can take. For the *i* ^th^ locus under haplogroup *g*, the bins for allele value *x* are given by
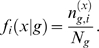
(13)As an example, a set of *L* densities for a haplogroup and how they are evaluated for a given sample are shown in Figure 8.

The distribution Equation 11 is defined over all haplogroups, but is not by itself a classifier. To make a decision, we choose the maximum under the posterior (MAP)
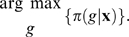
(14)This minimizes our risk of an incorrect classification. A benefit of the generative classifier is the ability to provide alternative classifications and a real probability associated with each decision.

### Support Vector Machines

Support vector machines learn a hyperplane with maximal margin of separation between two classes of data samples in a feature space [29],[30],[31]. We trained SVMs for binary haplogroup classification by treating locus alleles for a sample as *L*-dimensional vectors in Euclidean space and learning a hyperplane to separate them. A new sample is classified according to which side of the hyperplane its allele values fall on. We first address the case of deciding between two haplogroups to describe the standard support vector machine approach. We then introduce a method to combine binary classifiers into a multi-way classifier for all haplogroups using evolutionary evidence for haplogroup relationships.

For a sample **x** *_n_* of locus alleles, consider the task of deciding between two haplogroups with labels {−1,1}. If we assume the locus allele values between the two haplogroups are linearly separable in some feature space, we can use the classification model
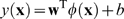
(15)where *y*(**x**) = 0 is a *L*-dimensional hyperplane separating the two haplogroups; *φ*(⋅) is any constant transformation of the allele values into a feature space. Thus, for the *n* ^th^ sample, the haplogroup is *g_n_* = 1 when *y*(**x** *_n_*)>0 and *g_n_* = −1 when *y*(**x** *_n_*)<0.

**Figure 9:**
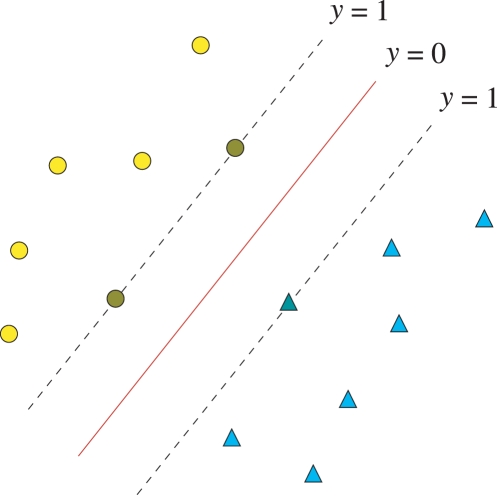
Maximum margin hyperplane used in support vector machines. Example showing the hyperplane with maximal margin of separation between samples from two different haplogroups. The shaded points lying on the margin define the support vectors.

The goal of training a support vector machine is to find the hyperplane, defined by **w**, *b* in Equation 15, giving the maximum margin of separation between the data points in the two haplogroups, Figure 9. The margin is defined as the smallest perpendicular distance between the separating plane and any of the data points in the sample.

By noting that the distance of a sample **x** *_n_* from the hyperplane is |*y*(**x** *_n_*)|/||**w**||, and that *g_n_y*(**x** *_n_*)>0 for all samples in the training data, then the maximum margin solution is described by the optimization
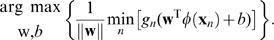
(16)However, solving this optimization problem directly is difficult, so we re-formulate it as follows.

Without loss of generality, we can rescale **w**, *b* so that the sample(s) 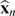 with allele values closest to the hyperplane satisfy
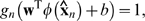
(17)as in Figure 9. Then the optimization problem Equation 16 reduces to maximizing ||**w**||^−1^, which is equivalently re-formulated for convenience as
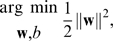
(18)with the constraint that *g_n_*(**w** ^T^ *φ*(**x** *_n_*)+*b*)≥1, for all 1≤*n*≤*N*. This can be solved as a quadratic programming problem by introducing Lagrange multipliers *a_n_*≥0 for each constraint, giving the function
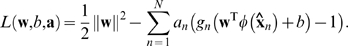
(19)By differentiating *L*(⋅)*L*(⋅) with respect to **w** and setting it equal to zero, we see that
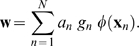
(20)Substituting the above into the classification Equation 15, we obtain
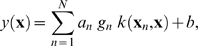
(21)where the kernel function is defined as *k*(**x** *_n_*, **x**) = *φ*(**x** *_n_*)^T^ *φ*(**x**). Therefore, training the model amounts to solving the quadratic programming problem to determine the Lagrange multipliers **a** and the parameter *b*. This is typically done by solving for the dual representation of the problem.

Transforming the problem into its dual shows that the optimization exhibits the Karush-Kuhn-Tucker conditions that
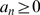
(22)

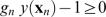
(23)

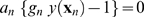
(24)Therefore, every sample in the training set will either have its Lagrange multiplier *a_n_* = 0, or *g_n_y*(**x** *_n_*) = 1. The samples whose multiplier is zero have no contribution to the sum in Equation 21, so they do not impact the classification. The samples that have non-zero multipliers are the support vectors and lie on the maximum margin hyperplanes, as in Figure 9; they define the sparse subset of data used to classify new samples.

A common and effective kernel to use for SVMs is the Gaussian, which has the form
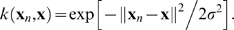
(25)We chose to use this kernel and assume the haplogroups are linearly separable in this transformed space over locus allele values.

In order to make the SVM approach work on data that may not be perfectly separable, we allow for some small amount of the training data to be misclassified. Thus, rather than having infinite error when incorrect (zero error when correct), we allow some of the data points to be classified on the wrong side of the separating hyperplane. To accomplish this, we follow the standard treatment of introducing slack variables that act as a penalty with linearly increasing value for the distance from the wrong side [30],[31].

A slack variable *ξ_n_*≥0 is defined for each training sample with *ξ_n_* = 0 if the sample is on or inside the correct margin boundary and *ξ_n_* = |*g_n_*−*y*(**x** *_n_*) if it is incorrect. So we now minimize
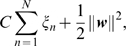
(26)where *ξ_n_* are the slack variables, one for each data point, and *C*>0 weights the significance of the slack variables to the margin in the optimization.

The optimization process is similar to before, but the Lagrange multipliers are now subject to the constraint 0≤*a_n_*≤*C*. As before, the samples whose multipliers are non-zero are the support vectors. However, if *a_n_* = *C*, then the sample may lie inside the margin and be either correctly or incorrectly classified, depending on the value of *ξ_n_*.

Since SVMs train a binary classifier and we have multiple haplogroups to distinguish between, we trained an SVM for each haplogroup in a one-vs-many fashion. In general, an SVM trained as one-vs-many for a particular haplogroup uses samples in that haplogroup as positive exemplars and samples in other haplogroups we wish to compare against as negative exemplars.

**Figure 10:**
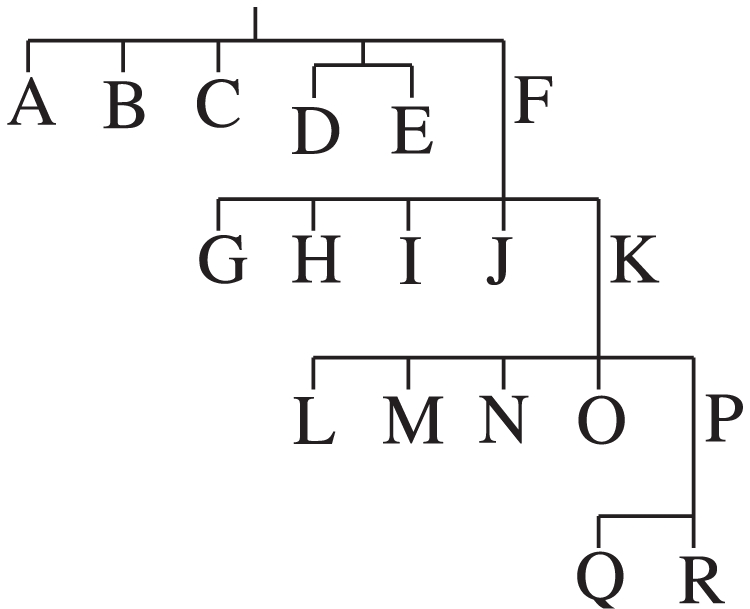
Y chromosome haplogroup hierarchy. Only the top-level haplogroups are shown.

We organized the set of binary classifiers into a hierarchy based on the currently known binary haplogroup lineage [12],[13]. At each level of the hierarchy, Figure 10, the one-vs-many classifiers are trained using only samples with haplogroups at that level, descendant levels, or ancestors; the samples at other branches are not used. Classification down the tree is accomplished by choosing the SVM result that has a positive classification. When there is more than one positive classification (or all negative), we choose the result with the closest distance to the support vectors. If the haplogroup the sample is best associated with is not a leaf node, it is further evaluated down the tree until a leaf is reached.

### Implementation

In order to create a high throughput classification system, all samples of STR data needing haplogroup prediction are batch selected from a database at regular intervals and classified. Once selected, our tandem classification software predicts the haplogroup for each sample and updates its record in our laboratory information management system (LIMS). Laboratory technicians and data reviewers can then view the results in a web interface (Figure S4) for the classified batch of samples. The LIMS displays which samples need to be SNP tested for haplogroup verification (based on lack of tandem agreement). Once verified, the tested samples are added to the ground-truth set to improve future classifications.

The tandem classification software brings together a collection of algorithms implementing naive Bayes, support vector machines, and decision tree classifiers. Where available, we used standard implementations of these algorithms that are open and available to the public.

For support vector machines, we used the freely available software package libSVM [32], which is written in C++. We added a customized extension to the library to support multi-class haplogroup prediction as previously described, where the set of trained one-vs-many binary SVM classifiers are organized into a hierarchy that follows Figure 10. In addition to providing training and binary classification algorithms, the SVM library provides tools to efficiently iterate over possible constants and kernel parameters using cross-validation in order to find the best set to use.

The decision tree classifiers J48 and PART were used as components of the Weka machine learning software suite [26]. The software is written in Java and called from our tandem classification software as an external program.

## Supporting Information

Figure S1 NRY haplogroup SNP tree used to type each sample in our ground-truth training set.
(0.02 MB EPS)

Figure S2 Accuracy of each classifier per haplogroup of predicting the public STR data set using the 9-locus subset of our ground-truth data as training. The lower panel shows the frequency of samples among haplogroups in the public data set.
(1.06 MB EPS)

Figure S3 Tandem classification accuracy and agreement per haplogroup on the public data set using the 9-locus subset of our ground-truth data as training.
(0.03 MB EPS)

Figure S4 Screen capture of the STR score review console from the laboratory information management system. The red dashed box contains STR loci names as headings for the columns of collected loci score data. The pop-up window (in black) show the quality assessment score for a selected sample. The yellow dashed ellipse highlights our software's haplogroup classification and confidence value for each algorithm in the tandem approach.
(0.44 MB EPS)
